# Adjustment of Sonar and Laser Acquisition Data for Building the 3D Reference Model of a Canal Tunnel †

**DOI:** 10.3390/s151229855

**Published:** 2015-12-11

**Authors:** Emmanuel Moisan, Pierre Charbonnier, Philippe Foucher, Pierre Grussenmeyer, Samuel Guillemin, Mathieu Koehl

**Affiliations:** 1 Cerema Dter Est, Image Processing and Optical Methods Research Team, 11 rue Jean Mentelin, B.P. 9, Strasbourg 67035, France; Pierre.Charbonnier@cerema.fr (P.C.); Philippe.Foucher@cerema.fr (P.F.); 2 ICube Laboratory UMR 7357, Photogrammetry and Geomatics Group, INSA Strasbourg, 24 Boulevard de la Victoire, Strasbourg 67084, France; pierre.grussenmeyer@insa-strasbourg.fr (P.G.); samuel.guillemin@insa-strasbourg.fr (S.G.); mathieu.koehl@insa-strasbourg.fr (M.K.)

**Keywords:** 3D modeling, terrestrial laser scanning, multibeam echosounder, robust estimation, registration of underwater data, LiDAR

## Abstract

In this paper, we focus on the construction of a full 3D model of a canal tunnel by combining terrestrial laser (for its above-water part) and sonar (for its underwater part) scans collected from static acquisitions. The modeling of such a structure is challenging because the sonar device is used in a narrow environment that induces many artifacts. Moreover, the location and the orientation of the sonar device are unknown. In our approach, sonar data are first simultaneously denoised and meshed. Then, above- and under-water point clouds are co-registered to generate directly the full 3D model of the canal tunnel. Faced with the lack of overlap between both models, we introduce a robust algorithm that relies on geometrical entities and partially-immersed targets, which are visible in both the laser and sonar point clouds. A full 3D model, visually promising, of the entrance of a canal tunnel is obtained. The analysis of the method raises several improvement directions that will help with obtaining more accurate models, in a more automated way, in the limits of the involved technology.

## 1. Introduction

The use of three-dimensional (3D) data acquisition systems (point clouds or images) for building models of partly-submerged infrastructures is currently undergoing an important development. In the literature, many systems, including industrial solutions, combine underwater and terrestrial sensors to investigate structures, such as dams, harbors or pipelines [1,2,3,4]. However, it may be noticed that only a small number of published works consider the accuracy assessment of the produced 3D models, by comparing them to some reference models [5,6,7].

**Figure 1:**
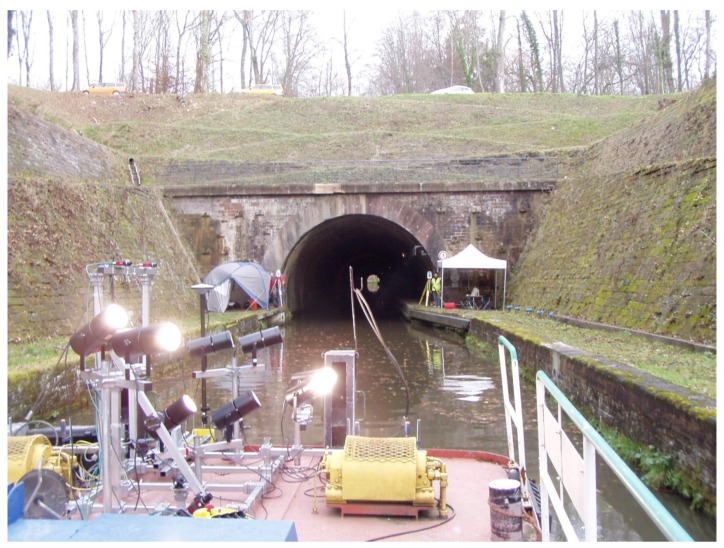
Modular on-board mobile image recording system at the experimental site.

**Figure 2:**
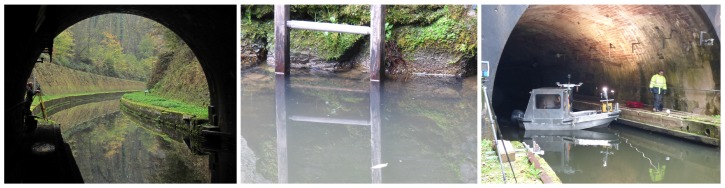
Constraints that apply to recording systems in tunnel canals. (**Left**) Global navigation data are not available in tunnels, nor at their entrances, because satellites are masked, hindering conventional mobile mapping; (**Center**) The turbidity of water prevents using optical imaging devices; (**Right**) The canal is shallow and narrow, so robust sonar processing algorithms are needed.

In this paper, we focus on the construction of an accurate 3D model of the entrances of a tunnel canal, from static acquisitions of point clouds. This model shall be used as a reference for future accuracy assessments in the context of the development of an embedded acquisition system devoted to the full 3D modeling of canal tunnels (*i.e.*, including both their underwater and above-water parts). Indeed, conventional mobile mapping systems cannot be used for positioning a barge, because global navigation satellite systems (GNSS) do not work, neither in tunnels nor at their entrances, which are most of the time bordered by narrow embankments (see Figure 1 and Figure 2-left), so innovative solutions must be proposed. Potential application concerns, in France, 31 tunnels currently in use, representing 42 km of underground waterways: the maintenance of these structures is a necessity, not only for preserving the historical heritage they represent, but also for protecting goods and persons. In the context of a partnership between Voies Navigables de France (VNF, the French operator of waterways), the Centre d’Études des tunnels (CETU) and the Cerema, in collaboration with the Photogrammetry and Geomatics Group at INSA-Strasbourg (institut national des sciences appliquées), an image acquisition prototype, embedded on a barge, has been devised for imaging the tunnel vaults and side walls (see Figure 1). During this project, solutions to geo-reference data precisely in the tunnel have been proposed and evaluated [8]. This system is going to be equipped with a multibeam echosounder to provide 3D views of the underwater parts of tunnel canals.

A 3D reference model will be necessary to assess the accuracy of the model of the whole tunnel provided by the mobile recording system under development. We have chosen to build this model from separate, static acquisitions of the under- and above-water parts of the tunnel entrances. For the above-water parts, point clouds have been collected using a 3D terrestrial laser scanning (TLS) system. A previous evaluation of the resulting 3D terrestrial model has shown that its accuracy is 1.7 cm [8]. Since the water turbidity (see Figure 2-center) excludes the use of optical sensors, the acquisitions of the underwater parts of the canal have been performed using a 3D mechanical scanning sonar (MSS) from static positions, in a similar way to a TLS [9,10]. This emerging technology may provide more accurate models than mobile systems. However, unlike the processing of TLS data, the registration and geo-referencing of MSS point clouds are complex, and several challenges need to be solved. MSS data are intrinsically noisy, and the narrowness and shallowness of the canal (Figure 2-right) induce artifacts due to water surface and sidewall reflections. Therefore, robust methods must be sought to alleviate these difficulties and reconstruct an accurate 3D model of the underwater part.

Aligning the 3D underwater model with the TLS one to form the global reference model is the second challenging problem, because, at every scanning position, the location and the orientation of the sonar device are not directly available. In this contribution, we propose to generate directly the full 3D model of the canal tunnel by co-registering the above- and under-water point clouds. Such an approach ideally requires an overlap between the TLS and MSS point clouds. In our case, there is no overlap, but we can exploit some geometric primitives (planes, lines, silhouette of the waterline), which are common to the above- and under-water parts of the structure. Moreover, we partially immersed wooden ladders on each canal bank. The robust fitting of the rungs and stiles of the ladders in both point clouds, using Maximum likelihood-type estimators (M-estimators), provides additional constraints for modeling the canal tunnel.

The paper is organized as follows. We first review related works in Section 2. Then, in Section 3, we introduce TLS and MSS data recording systems. Sonar data processing is described in Section 4. Section 5 is dedicated to the construction of the full 3D model by co-registering the above- and under-water models. In Section 6, we comment on the experimental results. Section 7 concludes the paper and proposes future directions for this work.

## 2. Related Work

For the purpose of assessing the accuracy of 3D acquisitions of civil engineering structures, the 3D reference model has to be more accurate than the model under inspection. Under certain circumstances, construction plans are available, and an as-built model may be used. For example, in [11], the evaluation of 3D reconstructions by an underwater SLAM (simultaneous localization and mapping) method was performed with a computer-aided design (CAD) model of a ship hull. Most tunnel canals, however, were bored during the 19th and 20th century (e.g., our test structure, Niderviller’s tunnel, located near Strasbourg (France), was bored between 1839 and 1845), and their construction plans, even limited to the headwalls, are not available or not accurate enough, so other solutions must be sought.

The alternative for building ground-truth models is to survey the object with a very accurate measurement device. For the above-water parts, geo-referenced points or point clouds may be used. In [6], the accuracy of data collected by a boat-based mobile laser scanning system is evaluated by using a set of reference spheres, positioned by GNSS. In [12], a set of geo-localized TLS point clouds provided reference data with centimetric precision for a mobile mapping system verification. A similar technique was reported in [8].

In the case of partly-immersed structures, there are two possibilities. The first one is to immerse an artificial reference object that can be surveyed beforehand, using a static TLS, for example. This technique was recently used in [7] to evaluate CIDCO (Centre Interdisciplianire de Développement en Cartographie des Océans) sonar prototypes. In this work, a test bench made of concrete panels with protrusions and extrusions was scanned by a TLS at a millimeter resolution, with a 2-cm resampling before being immersed and surveyed. The second one is to exploit existing structures that can be emptied, such as dry docks, or filled, such as dam reservoirs. For example, in [5], the Blueview company performed acquisitions in dry docks to evaluate a multi-beam sonar equipment. A TLS survey of the empty dry dock was first performed, and sonar acquisitions were made after filling the dock. In [7], a 3D LiDAR model of a dam was acquired before the reservoir was filled, and then, a survey was made using a multi-beam echosounder (MBES), showing only slight, local differences (less than 5 cm) due to the shift of materials during the filling operation. In a similar manner, it is possible to benefit from natural effects, such as tide. For example, in [1], a surface was surveyed at high tide using a sonar system, and a total station was used to assess the acquisition process at low tide.

**Figure 3:**
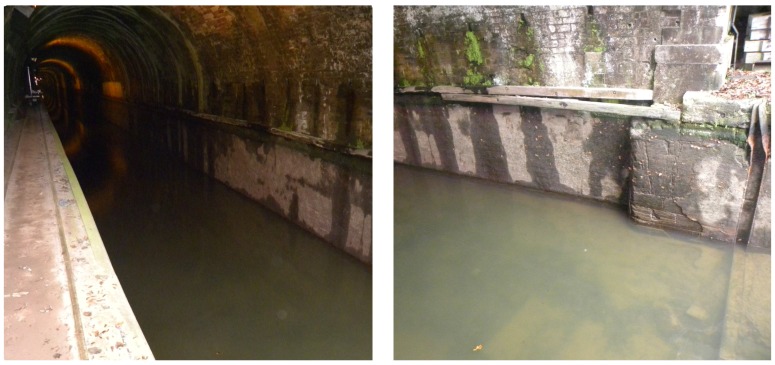
Entrance of Arzviller’s tunnel during its emptying in 2009.

In our case, it would have been interesting to complement the existing TLS survey of the above-water parts of our test structure, Niderviller’s tunnel, after emptying the canal. Unfortunately, such an operation is costly and may even be hazardous due to the age of the tunnel: the canal walls, in poor condition, might crumble. Therefore, canal managers are often reluctant, and drying operations occur very rarely. For example, Niderviller’s tunnel and a nearby one, Arzviller’s tunnel, were emptied in 2009 (before the beginning of our study); see Figure 3. This was the first time since 1968. Since it is not possible to empty the canal, we have to resort to 3D underwater imaging techniques. Mobile or static systems might be envisioned.

In recent years, mobile surveying systems were developed to inspect partially- or totally-immersed open structures, like harbors or dams. Most of these operational systems provide 3D models thanks to MBES for the underwater parts and terrestrial laser scanner (TLS) for out-of-the-water parts. The acquisition is performed in a dynamic way in order to sweep the surveyed structure. The localization of the system is hence of central importance. Most of the time, the associated positioning system combines GNSS and inertial navigation systems (INS); see, e.g., [1,3]. However, these methods are unsuitable for canal tunnels due to the lack of a GNSS signal. Alternatives to GNSS/INS systems were proposed in the robotics and computer vision literature. For example, [13] introduces a SLAM approach to obtain at the same time the 3D model and the mobile localization, thanks to the registration of TLS point clouds acquired with high frequency. In [8], we proposed a simplified visual odometry technique to estimate the position of our mobile mapping prototype along the tunnel. However, all of these methods suffer from drifts that are especially sensible in elongated structures, such as tunnels, and that can only be corrected using reference points, which are more difficult to set up in the absence of a GNSS signal. Other potentially interesting systems were developed in the context of large-diameter pipe inspection. Recently, two devices were proposed: the ABIS (above and below inspection system) system of the ASI Marine Technology society (see [4]) and the HD Profiler System of the Hydromax USA society (see [2]). Both systems acquire laser and sonar data and HD images. The documentation of these commercial solutions is limited. Furthermore, a towed device cannot be used in certain tunnels, which are curved.

In order to avoid the difficulties related to the localization of mobile mapping systems and to obtain an accurate reference model, we choose to perform static acquisitions to build the underwater part of our reference model. Such an approach is classical for TLS data. Thanks to the recent availability of a 3D mechanical scanning sonar (MSS), it is now possible to get a 3D model from sonar acquisition in a static way, as well. The MSS device was used in several underwater surveys, such as the ones introduced in [9,10]. In a TLS-like manner, several scanning positions were carried out to get a full model. The co-registration of point clouds was done, in a local coordinate system, with the iterative closest point (ICP) algorithm.

The positioning of underwater cloud points in the same coordinate system as the TLS data can be performed in direct or indirect ways (see [14]). Both methods require the knowledge of reference points. In the literature, one may find several solutions to define these points underwater. The first method is acoustic positioning based on triangulation. The operating principle is close to GNSS, except, in the water, acoustic waves are used. For example, in [15], buoys equipped with ultra-short baseline (USBL) transceivers, tied up with GNSS receivers, yield the localization of a remotely-operated vehicle (ROV). When the survey is carried out in shallow water, underwater points can be surveyed with terrestrial methods thanks to a long pole equipped with a prism for a total station or a GPS antenna (see [16]). Lastly, in [17], poles with underwater and above-water targets are partially immersed. Thus, under- and above-water acquisitions can be co-registered, because poles create a link between both models. In the present work, we have chosen a similar solution, in which wooden ladders, as well as existing geometric primitives of the structure are used to link the underwater and above-water models.

## 3. Data Recording

In this section, we introduce the setup that was used to build the 3D reference model of the canal tunnel on the site of Niderviller using two up-to-date scanner devices.

### 3.1. Scanner Devices

**Figure 4:**
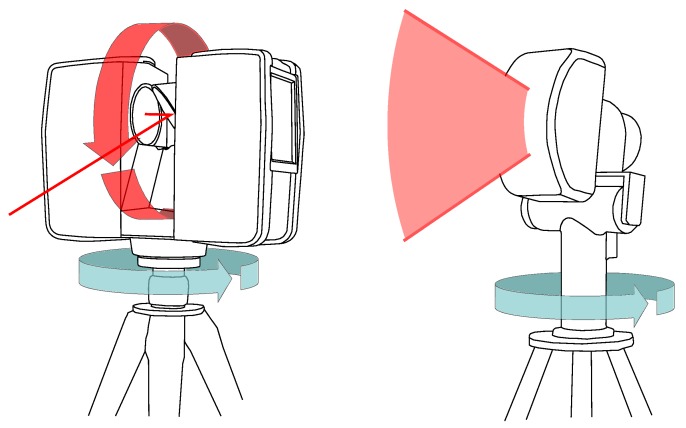
Schematic representation of acquisition sensors: The Faro${}^{®}$ (Lake Mary, FL, USA) Focus 3D X330 TLS (**Left**); The Blueview${}^{®}$ (Bothell, WA, USA) BV5000 mechanical scanning sonar (MSS) (**Right**).

**Figure 5:**
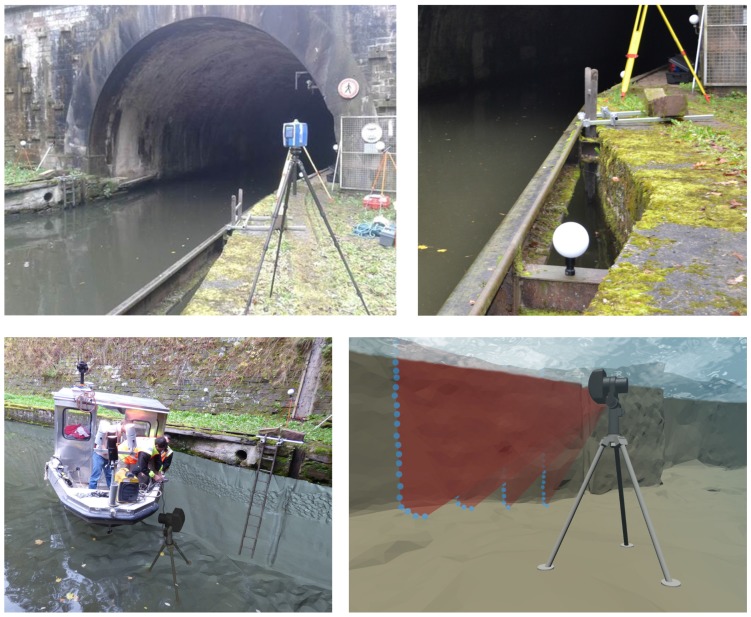
Data acquisitions in Niderviller’s tunnel: Photograph of the TLS device (**Top-left**); Zoom on a spherical targets and a ladder fixation on the dock side (**Top-right**); Schematic representation of MSS device positioning (**Bottom-left**) and Rotary scanning (**Bottom-right**).

The above-water acquisitions were performed using a Focus 3D X330 TLS, the latest Faro${}^{®}$ scanner; see Figure 4 (left), and Figure 5 (top-left). To survey the environment in 3D over a range of 0.6–300 m, a laser beam sweeps the visible surfaces, vertically and horizontally over almost 360°. The distance between the TLS and the objects is measured using the phase difference technique [14].

The underwater acquisitions were carried out with the Blueview${}^{®}$ (Bothell, WA, USA: www.blueview.com) BV5000 MSS (see Figure 4 (right) and Figure 5 (bottom)) operated by a sub-contractor, the Sub-C Marine company. The device is made of a multi-beam echo-sounder with a vertical swath direction and a rotation system with a vertical axis that enables a 360° horizontal scan (see Figure 5 (bottom-right)). Since the swath aperture is 45°, a mechanical system is used to tilt the sensing head, so potentially, a 320° vertical range can be scanned. We used three different tilt angles (0°, −9°, −30°) for this experiment. The acoustic sensor emits a high frequency signal (1.35 Mhz), which offers a good resolution in distance, but at the same time, limits the maximum acquisition range to 30 m. According to the manufacturer’s data sheet, the vertical and horizontal spatial resolution (*i.e.*, the distance between a recorded point and its closest neighbor) at 10 m are 16 mm and 30 mm, respectively.

The comparison between MSS and TLS features (see Table 1) highlights differences that will impact the relative quality of the recorded point clouds. For example, we can note a large difference in spatial resolution that will affect the point densities. However, the main difference concerns the beam width, which has a direct impact on the ability to distinguish two echoes coming from two different targets. For example, the signal footprint on a plane perpendicular to the signal direction 10 m away from the device is a circle of 6 mm in diameter for the Focus 3D X330 and a square of a 175 mm side length for the BV5000.

Note that all differences between point clouds are not imputable to the characteristics of the devices only. For example, the footprint size depends not only on the recorded object to the scanner, but also on the incidence angle of the signal, as we will see in Section 3.3.

**Table 1:** Manufacturer’s specification of the acquisition devices (top lines) and recording resolutions used for the experiment (two bottom lines).

	Faro Focus 3D X330	Blue View BV5000
beam width	2.25 mm + 2×0.011°	1°/1°
ranging error	±2 mm (10–25 m)	15 mm
maximum range	330 m	30 m
field-of-view (vertical/horizontal)	300°/360°	45°/360° (320°/360°)
horizontal resolution	0.035° (6 mm at range 10 m)	∼ 0.09° (16 mm at range 10 m)
vertical resolution	0.035° (6 mm at range 10 m)	0.18° (30 mm at range 10 m)

### 3.2. Experimental Setup

The experimental acquisitions have been carried out in Niderviller’s canal tunnel (see Figure 1). It is straight and lined with stonework. The radius of the vault is 4 m; the width of the canal is 6.6 m; and its water depth is about 2.5 m. It has a pedestrian path on a ledge that was formerly used as a towing path. About 7000 ships, among which a vast majority are pleasure boats, cross the tunnel annually (according to 2012 statistics). Albeit that the acquisitions took place during a low-traffic period, the recording time was constrained, and it was not possible to scan the full length of the tunnel (475 m). Therefore, the acquisitions were focused on its entrances, for this first experimental campaign.

At each entrance, two laser scans have been performed, from each bank of the canal, simultaneously with the underwater acquisition. The resulting model can be complemented using a previously-performed TLS survey of the whole tunnel. To register point clouds, spherical targets have been placed in the shared scanning area, as shown in Figure 5, top. In order to geo-reference the model, the coordinates of sphere centers have been established with traditional surveying methods based on a set of reference points implemented on site, in the French reference coordinate systems RGF 93 (réseau géodésique français) and NGF-IGN 69 (nivellement général de la France operated by l’Institut National d’Information Géographique et Forestière).

**Figure 6:**
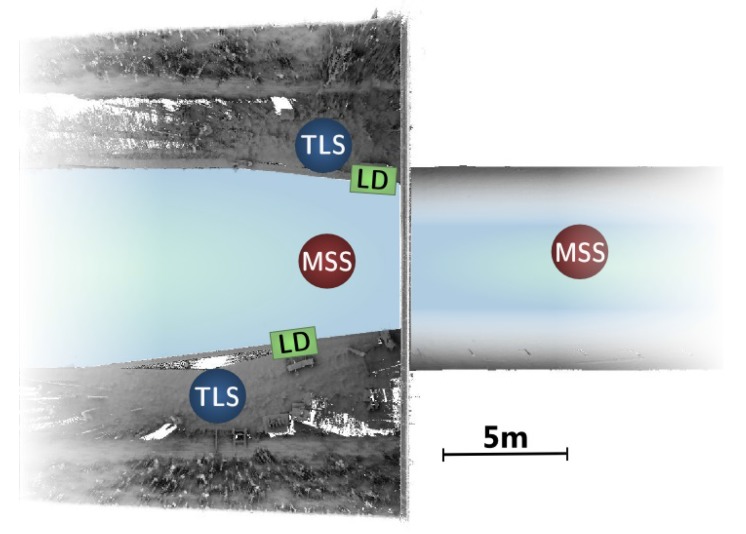
Illustration of the acquisition setup seen from above, featuring TLS and MSS scanning positions, as well as the location of ladders (LD).

Two sonar scans, from positions placed 10 m away from each other, have been performed on each entrance of the tunnel, one inside and one outside of it; see Figure 6. The MSS is attached to a tripod, which is sunk in the canal from a boat, as shown on the bottom left part of Figure 5. The soil consists of a mixture of rocks and mud. It proved to be sufficiently hard to support the weight of the tripod, which kept stable during all acquisitions (accidental motions of the instrument may be detected at the closure of a scanning rotation). Setting up the MSS took about 15 min by scan. The spatial resolution was adjusted, so the acquisitions themselves lasted 10 min by rotation. Three acquisitions, with different tilt angles, were used. The quality of the obtained signals was checked on site. Overall, each acquisition required about one hour.

Two wooden ladders (3.60 m high and 0.32 m wide) were partly immersed in the water, so they were recorded by both the MSS and the TLS. Ladders are ordinary objects, easily available and easy to set up on site, that are well suited to the application. Their length and geometric characteristics (parallelism, orthogonality, known inter-rung distances) help the registration of the underwater and above-water models (see Section 5). Wooden ladders were preferred to metallic ones because they yield cleaner sonar echoes. The ladders are weighted and attached to the dock side using a weighted frame, as shown in Figure 5 (top-right).

### 3.3. Remarks

A few remarks can be made in relation to the acquisition context. Of course, unlike TLS acquisitions, the underwater recording cannot be supported by visual control. To make a decision about the scanning positions, for example, we could only rely on above-water elements, which raises issues for both recording and interpreting sonar data.

**Figure 7:**
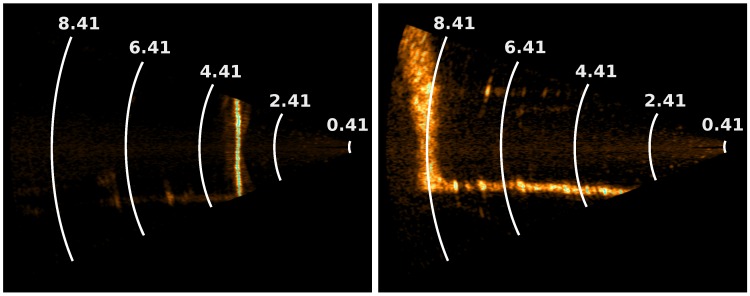
Multi-beam echo-sounder swaths under two different incidence angles. (**Left**) Almost perpendicular incidence (see the red line in Figure 8); (**Right**) Grazing angle (see the green line in Figure 8). The swath is wider in the second case. Distances are given in meters.

**Figure 8:**
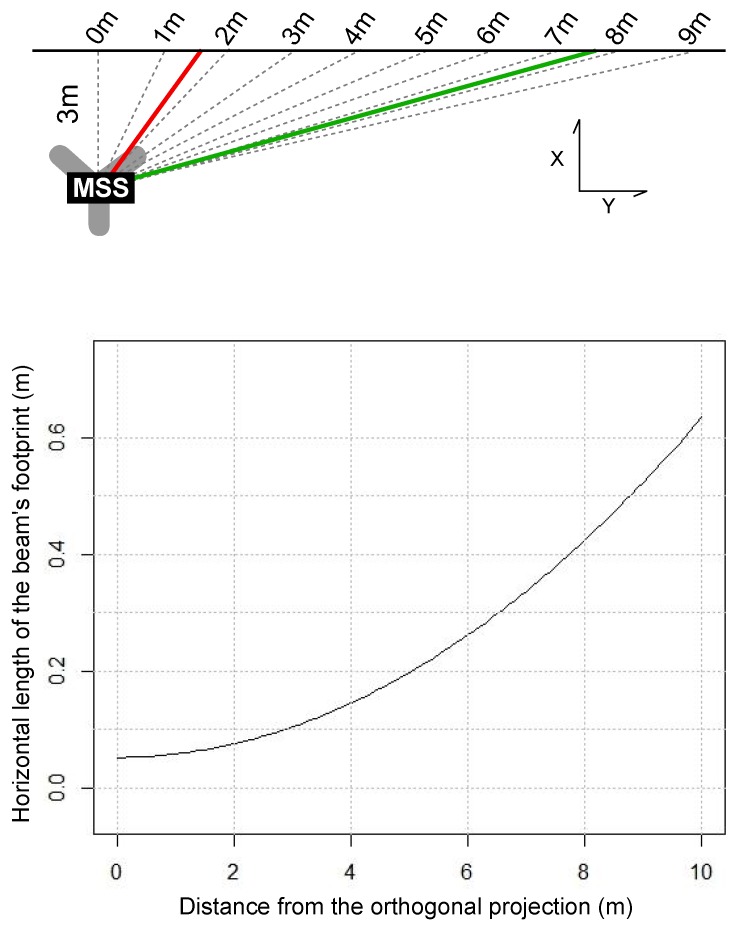
Top view of the theoretical acquisition setup (**Top**); Estimation of the sonar horizontal footprint size as a function of the distance from the orthogonal projection (**Bottom**).

Another remark is that the elongated shape of canal tunnel yields unfavorable incidence angles for sonar acquisition. This influence is visible in the acoustic images, shown in Figure 7. One can see that the vertical line, which corresponds to the footprint of the swath on the sidewalls of the canal, is much wider for a grazing incidence than for an almost perpendicular acquisition. According to a theoretical model of the acquisition setup and to the datasheet of the BV5000, we may estimate the horizontal width of the beam footprint (see Figure 8) by intersecting the emission beam model with a plane representing the sidewall. We see that, in the case of canal tunnels, this length can easily reach more than 0.5 m.

## 4. Sonar Data Processing

The first observation of raw sonar data highlights measurement errors and also the noisy nature of MSS point clouds. These elements must be handled. Furthermore, to obtain a full 3D model, underwater and above-water point clouds have to be adjusted by registration.

### 4.1. Sonar Measurement Errors

The raw sonar output is an acoustic image constructed from the received echoes in each beam. More specifically, from each beam, the echo with the highest intensity is used to estimate the 3D point. However, the acoustic image brings out some measurement errors. In most cases, these errors have a lower intensity than the recorded object, so they do not appear in the output point cloud, but some artifacts may remain.

Some measurement errors in sonar data may come from the scanner device itself. A list of error sources is reported in [5], along with their consequences on data recording and possible adjustments to alleviate them. Typical problems that may occur are: platform motion, tilt offset errors, insufficient coverage or incorrect sound speed. Some corrections can be applied in post-processing, but in some cases, new scans need to be taken. These rough errors are checked immediately after scanning. However, slight errors may be insensible in the raw point cloud.

**Figure 9:**
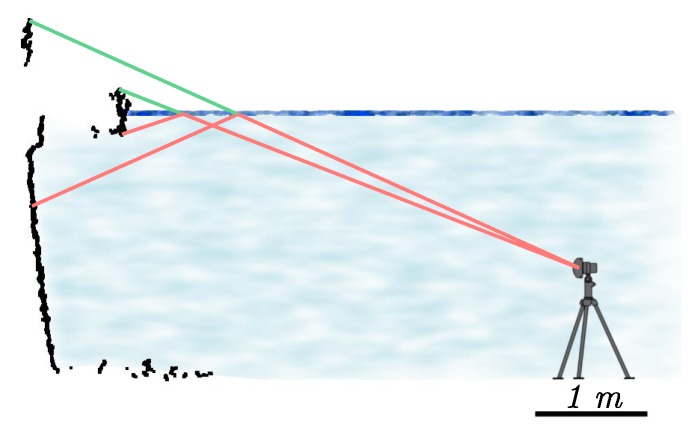
Signal reflection on the water surface may cause artifacts.

Other errors are due to the configuration in which the MSS scans are operated: shallow water and confined environment. The most visible errors are due to signal reflections on the water surface. In some cases, “phantom” objects may be observed above the water surface (see Figure 9). However, such reflections can be detected by visual inspection of the profiles, and the artifacts are then easily deleted from the point cloud. It is more difficult to detect surface reflections when the sidewalls of the canal are planar. They make the vertical position of the waterline more difficult to estimate. This is another justification of using ladders to help with geo-referencing the underwater point cloud. Once the MSS vertical position is known, surface artifacts can be deleted. Reflections may also occur on the sidewalls or on the raft. In the latter case, phantom objects are observed underneath the soil level, so they can be discarded.

Furthermore, some errors unavoidably arise due to the presence of objects in the canal, such as the boat hull, cables, fishes or suspended particles. All of these errors are deleted manually, but most of them could be removed automatically, because the canal shape is roughly known.

### 4.2. Denoising and Meshing

The first processing step consists of removing the significant noise exhibited by sonar acquisitions. The proposed solution exploits the meshing phase of the reconstruction process. Indeed, most of the time in surveying applications, surface reconstruction is performed to obtain a simplified digital model of the recorded structure, and this operation is generally the last step in the modeling chain. Here, we use it as a pre-processing step, since it provides a visual control on the result, which highlights errors and guides denoising.

**Figure 10:**
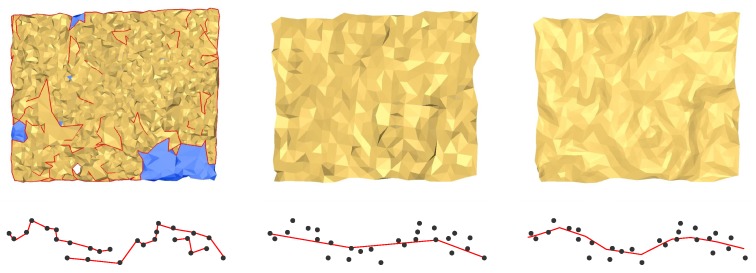
Methods for meshing point clouds: Using all points (**Left**); Using selected points (**Center**); Meshing using interpolated points (**Right**).

We note that TLS point clouds have a negligible noise level, so their processing only involves outlier removing, and all points may be employed as triangle vertices to reconstruct the surface. This is not the case for MSS clouds, for which artifacts may occur when all points are used for meshing; see Figure 10 (left). Removing those artifacts reduces to denoising and can be done using two methods:

- One is to select evenly-spaced points as mesh vertices for triangulation (Figure 10 (center)), based on a minimum distance criterion. The price to pay is that details may be lost.
- Another way is to compute the nearest surface to points using robust estimators (Figure 10 (right)). For this purpose, new points are interpolated. However, the risk is to obtain an over-smoothed model.

Meshing of point clouds may be carried out using specialized software; see, e.g., [18] for a review. We use 3DReshaper${}^{®}$ (Genay, France: www.3dreshaper.com), which has the ability to mesh with both previously-mentioned techniques and also to combine them to perform successive refinements of the model using the point cloud. Thus, the underwater model reconstruction is performed in a coarse-to-fine manner. The process starts with a large-scale mesh made by selecting points according to a minimum distance criterion. Then, points are picked again in the cloud or computed by interpolation to progressively increase the mesh resolution. Point selection involves either a distance-to-mesh or a maximum surface deviation criterion. The parameters are empirically tuned by an operator, and the process requires a trade-off between details and noise.

We note that this step of the process requires many manual operations, like outlier removal or correction of mesh reconstruction mistakes. While such interventions may be supported by photographs or other physical measurements for TLS data, this is not the case for underwater data, except in particular situations. Hence, the construction of the underwater model from MSS data involves an important part of interpretation.

**Figure 11:**
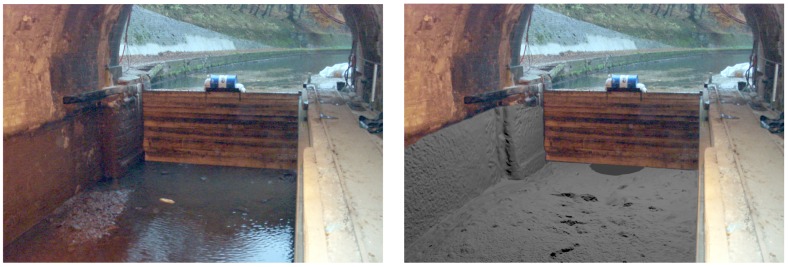
(**Left**) Photograph on the northern tunnel entry, during its emptying for maintenance in 2009 (source: Voies Navigables de France (VNF) archives); (**Right**) Visualization of the MSS denoised model (in grey) superimposed on the image. The orientation is done manually, thanks to the masonry block and the cofferdam grooves, which are visible in the model.

However, the example in Figure 11 shows that visually-correct results may be achieved using this method. We note that the filtered MSS surface shown on the rightmost part of the figure was obtained without any knowledge of the underwater structure: the photograph was found in VNF archives after the experiment.

### 4.3. Underwater Registration

The co-registration of MSS data aims at gathering all records in a single point cloud corresponding to the underwater part of the tunnel. For this purpose, the position between scans must be estimated. Since, in our setup, the position and orientation of the underwater scanner cannot be directly measured, we have to resort to the indirect method. Cloud-to-cloud registration seems to be the easiest technique to implement, but it also raises several issues. Some are due to the nature of the technology itself: MSS data are very noisy, and the resolution is rather coarse, so finding correspondences is difficult. Other difficulties are related to the elongated shape of the canal and to our experimental setup: the farther the recorded point, the smaller the grazing angle. Therefore, points in overlapping zones have a poor accuracy, which also influences the quality of registration. In these conditions, it is very difficult to solve the longitudinal ambiguity, *i.e.*, to accurately estimate the translation along the tunnel axis. Immersing geometric reference objects (e.g., ladders) or decreasing the distance between scanning positions to increase the overlap quality are possible solutions to this problem.

## 5. Model Alignment and Geo-Referencing

In this section, we introduce the method we propose for registering sonar data on laser data to form the full 3D reference model. We recall that this model will be used for assessing the accuracy of the models provided by a mobile mapping system for canal tunnels, currently under development. Comparing 3D models can be done in any arbitrary coordinate system. However, our test site is equipped with a geodetic model, so the model can be geo-referenced without additional complexity.

### 5.1. Registration and Geo-Referencing Method

In general, there are two ways of registering and geo-referencing point clouds [14]. The first one is direct: it requires the knowledge of the position of the scanner. The latter can be obtained *a priori*, by placing the device at a point of known coordinates, or *a posteriori*, by surveying its position using conventional techniques, but both solutions are difficult to put into practice for underwater acquisitions. The second one is indirect, *i.e.*, point clouds themselves are used to be registered and geo-referenced. Registration and geo-referencing of point clouds can be based either on targets or on clouds.

Target-based registration requires anticipating and placing targets in the field of view. Their geometry and scale depend on the spatial resolution and precision of the scanner. Spheres are usually used for TLS recording because the determination of their centers can be made very accurately. Of course, the quality of the registration also depends on the distribution and number of targets. Furthermore, when sphere centers are known in coordinates in a defined system, point cloud geo-referencing can be deduced straightforwardly. In our application, we use this method, which is very usual in laser scanning, for geo-referencing the TLS point clouds and form the above-water part of the model. Spherical open frames, proposed by the Blueview company (patent pending), may also be used as targets for MSS data; see [5]. No such targets were available for our acquisition campaign, but we experimented with ladders instead.

In general, cloud-based registration algorithms involve two steps. First, homologous points between scans are found. Then, these correspondences are used to estimate the geometric transformation (rotations, translations) between sets of points. The most popular algorithm is the ICP method, introduced by [19], which iterates these steps to minimize the discrepancy between the first set of points and the geometric transformation of the second set of points. This method requires a certain overlap between point clouds and, also, a first estimation of the transformation. An alternative technique is based on the detection of homologous geometrical entities between scans and finding the best way to align them. These entities can be planes, spheres, cylinders or lines. As for target-based methods, geo-referencing of the point cloud is a by-product of the registration step if the coordinates of some of the features are known.

Four observations can be made that form the basis of the proposed registration and geo-referencing method:

- some elements of the recorded scene can be approximated by geometrical entities, and certain ones of these are surveyed at the same time by the underwater and terrestrial scanners;
- targets (ladders) create a link between both environments;
- the projection of the waterline on the structure is the only contact element between both models;
- the silhouette of the waterline features many salient elements that can be used to align both models in a horizontal plane.

**Figure 12:**
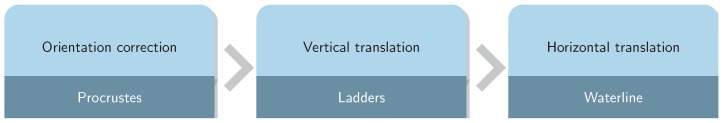
Flow chart of the registration of a sonar point cloud on a laser point cloud.

Following the above remarks, we implement a three-step registration method, depicted in Figure 12. First, the orientation angles are corrected thanks to the Procrustes method [20] using common geometric primitives. Then, the fitting of ladders on both under- and above-water point clouds enables estimating the vertical translation vector. Last, the 2D silhouette of the waterline is extracted from both models to estimate the horizontal translation.

### 5.2. Orientation Correction

**Figure 13:**
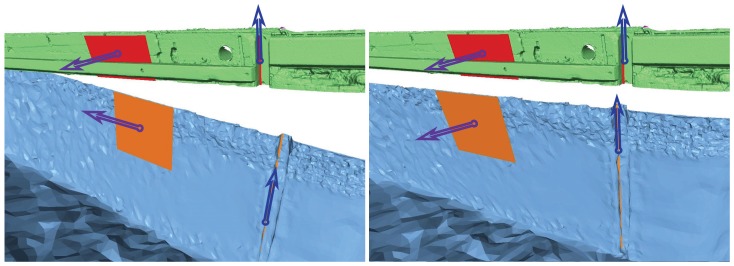
Original orientation of the TLS (in green) and the MSS (in blue) models (**Left**); Aligned models (**Right**).

The first step aims at correcting the orientation angles of the underwater model. To this end, common geometrical entities are manually fitted on certain parts of both models. For example, canal banks are surveyed under and above the water and approximated by planes. The normals to these common planes should be collinear. Using several such normals, the underwater model orientation can be computed, in the form of a 3×3 rotation matrix, Q. Other primitives, such as the directions of salient lines (e.g., cofferdam groove corners; see Figure 13) can also be taken into account: a minimum of two non-collinear vectors are required to estimate the rotation matrix.

A property of the rotation matrix that must be taken into account in the computation is its orthogonality. To perform the alignment, we use the solution described in [20] to the so-called “orthogonal Procrustes problem”:

(1) $$\min∥A-BQ{∥}_{F}^{2}\;\mathrm{subject}\;\mathrm{to}\;Q^{T}Q=I$$

where $∥.{∥}_{F}$ denotes the Frobenius norm and where A and B are, respectively, the direction vectors of the elements extracted from the TLS and the MSS models. The algorithm computes the singular value decomposition (SVD) of the $B^{T}A$ product, $U^{T}\left(B^{T}A\right)V=\Sigma$, to get the rotation matrix Q, as:

(2) $$Q=UV^{T}$$

Once Q is estimated, the orientation of the underwater model can be corrected; see Figure 13 (right).

### 5.3. Vertical Translation

**Figure 14:**
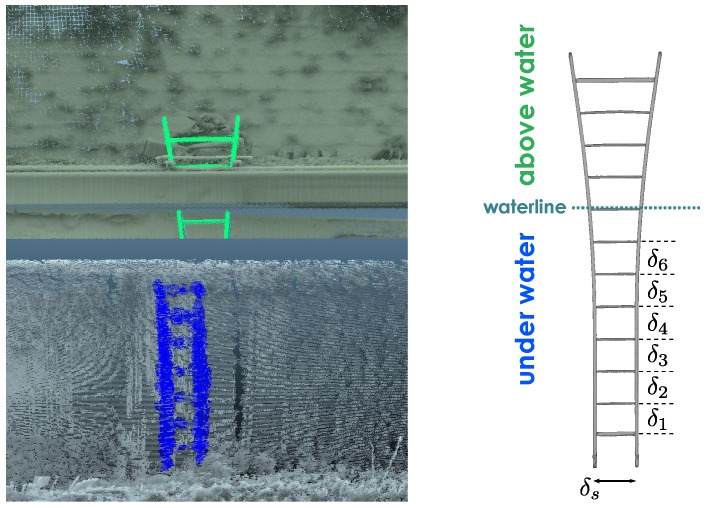
(**Left**) Segmentation of a ladder in the TLS model (**Top**, in green) and in the MSS model (**Bottom**, in blue). The upper part of the ladder is partly masked because it was placed behind a boom, (**Right**) Reference TLS survey of the ladder.

After the orientation of the underwater model has been corrected, the next step is to correct the vertical translation between models. For this purpose, we use the information provided by ladders that were immersed before the acquisitions, in such a way that they are visible in both point clouds; see Figure 14.

#### 5.3.1. Basic Ideas

The principle of the method is to use the distance between the rungs of the ladders to compute the vertical difference between models. These inter-distances, denoted by $\delta_{i}$, are given by a reference TLS survey. Figure 14 (right), shows the ladder that was used in this experiment. It is straight in its lower part and flared at the top. It was placed behind a protection rail (boom) during the survey, so its upper part is partly masked in the TLS point cloud (Figure 14 (left)). Note that one rung, that was just below the water surface, was not used. Indeed, only a few points were distinguishable in the point cloud due to surface reflection artifacts. This way, only the straight part of the ladder is used.

**Figure 15:**
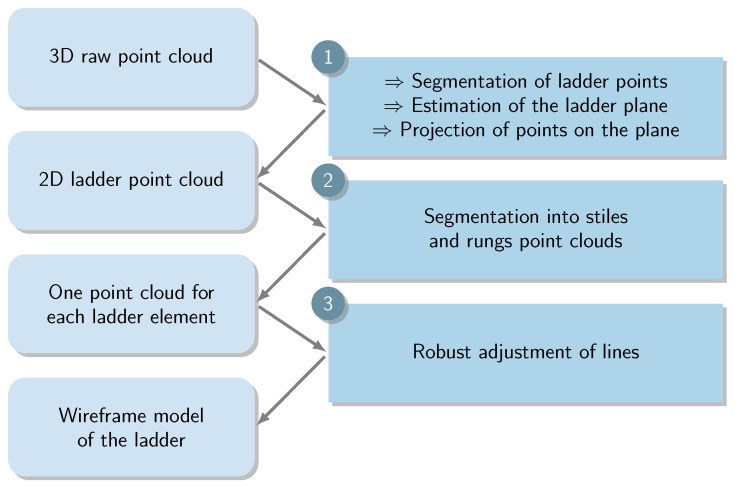
Flow chart of the ladder adjustment method.

**Figure 16:**
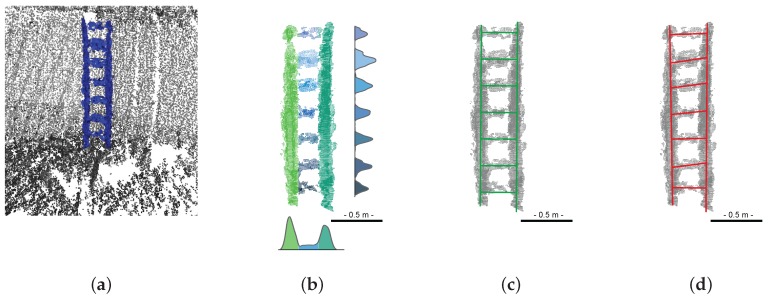
Illustration of the ladder fitting process: Extraction of ladder points from raw data (**a**); Segmentation into separate point clouds (**b**); Robust fitting of ladder using M-estimation and structural priors (**c**); Non-robust fitting using commercial software (**d**).

In order to estimate the vertical gap between models, it is first necessary to adjust a set of lines on both ladders in the point clouds. The proposed method is split into three steps, as described on Figure 15 and illustrated in Figure 16a–c.

While the TLS point cloud is not very noisy, there are many outliers in the MSS point cloud (Figure 14 (left)). This makes the extraction of the lower part of the ladder a rather challenging task, which requires the use of robust regression techniques. In this paper, we use M-estimators [21], which, in place of the usual sum of squared residuals, minimize a function of the form:

(3) $$J\left(\theta \right)=\sum_{i}\rho \left(r_{i}\right)$$

where *θ* is the vector of model parameters, *ρ* is a non-quadratic potential or penalty function and $r_{i}$ is the residual, *i.e.*, the difference between the observation and its prediction by the model. In the half-quadratic framework (see, e.g., [22]), it is shown that minimizing *J* is equivalent to minimizing:

(4) $$J^{⋆}\left(\theta ,b\right)=\sum_{i}b_{i}r_{i}^{2}+\Psi \left(b_{i}\right)$$

where Ψ is a convex penalty whose expression can be related to *ρ* and $b_{i}$ is an auxiliary variable, whose role is both to down weight outliers and to linearize the estimation problem. Indeed, $J^{⋆}$ is quadratic with respect to *r* (hence, for *θ* in linear regression) when *b* is fixed. It is convex with respect to *b* when *r* is fixed, and the minimum is obtained for $b=\rho^{\prime }\left(r\right)/2r$. Such properties suggest a deterministic algorithmic strategy that consists of alternately fixing each variable and minimizing with respect to the other. The resulting algorithms are iterative and perform a series of weighted least-squares (LS) estimations; see Equation (4). The weights *b* are adjusted at each iteration according to the value of the residuals. Moreover, $\rho^{\prime }\left(r\right)/2r$ is a decreasing function, such that b≃1 for small residuals and b→0 for large residuals. Hence, inliers are considered as in ordinary LS, while outliers are given a small weight, which reduces their influence on the estimation.

#### 5.3.2. Robust Projection and 2D Segmentation

The first step of the method is to manually extract the ladder points $x_{i}$ (for i=1,...,N) from the MSS point cloud. Then, we suppose that this set of 3D points may be approximated by a plane passing through an origin ***μ*** and spanned by two orthogonal unitary vectors $e_{1}$ and $e_{2}$ ($∥e_{1}^{⊤}e_{2}{∥}^{2}=0,∥e_{1}{∥}^{2}={∥e_{2}∥}^{2}=1$):

(5) $$x_{i}≃\mu +Ea_{i}$$

where $E=(e_{1},e_{2})$ and $a_{i}$ are the coordinates of the orthogonal projection of $x_{i}$ on the plane, which are given by $a_{i}=E^{T}\left(x_{i}-\mu \right)$. In the LS framework, the *i*-th residual is given by $r_{i}=∥\mu +Ea_{i}-x_{i}∥$, and the parameters of the model, namely the origin and the basis vectors, are estimated by minimizing:

(6) $$J_{LS}\left(\mu ,e_{1},e_{2}\right)=\overset{N}{\sum_{i=1}}{∥\mu +Ea_{i}-x_{i}∥}^{2}$$

The solution of this orthogonal regression problem (which is akin to principal component analysis, or PCA; see, e.g., [23]) is, for the origin:

(7) $$\mu =\frac{1}{N}\sum_{i=1}^{N}x_{i}$$

*i.e.*, the sample mean of the point cloud, and for the basis vectors, the solution of:

(8) $$CE=\lambda E\;\mathrm{with}\;C=\frac{1}{N}\sum_{i=1}^{N}\left(x_{i}-\mu \right){\left(x_{i}-\mu \right)}^{⊤}$$

In other words, the basis vectors are given by the two eigenvectors of the sample covariance matrix C that correspond to its two largest eigenvalues, $\lambda_{1}$ and $\lambda_{2}$. In the robust half-quadratic framework, the augmented energy is given by:

(9) $$J^{⋆}\left(\mu ,e_{1},e_{2},b\right)=\sum_{i}b_{i}{∥\mu +Ea_{i}-x_{i}∥}^{2}+\Psi \left(b_{i}\right)$$

The optimization of $J^{⋆}$ is performed by an iterative reweighted PCA algorithm. Each iteration alternates between: computation of the auxiliary variables $b_{i}=\rho^{\prime }\left(r_{i}\right)/2r_{i}$ (with $r_{i}=∥\mu +Ea_{i}-x_{i}∥$, for i=1,...,N), computation of the weighted mean:

(10) $$\mu =\sum_{i=1}^{N}b_{i}x_{i}/\sum_{i=1}^{N}b_{i}$$

and diagonalization of the weighted covariance matrix:

(11) $$C=\sum_{i=1}^{N}b_{i}\left(x_{i}-\mu \right){\left(x_{i}-\mu \right)}^{⊤}/\sum_{i=1}^{N}b_{i}$$

The complete algorithm is given in Appendix A. Once the origin and basis vectors are estimated, all points of the ladder cloud are projected onto the plane, and the rest of the process is performed in two dimensions. To avoid introducing new notations, data points will be denoted by $x_{i}$ thereafter, but from now on, they designate 2D projections in the ladder plane.

The robust plane estimation provides axes that follow the stile and rung directions quite well. Then, the distribution of coordinates along both axes shows peaks that correspond to ladder elements. These distributions, shown in Figure 16b, are approximated using Parzen-window density estimation with Gaussian kernels (see, e.g., [23]), at two different resolutions. The rough location of peaks is determined using a kernel resolution (bandwidth) of 1 cm. Then, the intervals between peaks are analyzed using a bandwidth of 1 mm: the list of local maxima of the distribution is iteratively filtered by non-maximum suppressions, until zero or one local minimum remains in the list. This analysis follows the spirit of the fine-to-coarse histogram analysis technique proposed by Delon *et al.* [24]. If no secondary peak exists within the interval, then the limits of the distribution are set by traversing the list of local minimum from the extremities of the interval until the distribution goes below an arbitrarily small value. Otherwise, the threshold is set at the first local minimum that goes below the height of the secondary peak. This method is successively applied to the horizontal and vertical coordinates, so the stiles are first separated from the ladders, then the rungs are extracted individually. Many other thresholding techniques might be used, but this one is rather simple and gives satisfactory results. Moreover, the segmentation stage is not a very sensitive step, since the estimation of the ladder model is performed in a robust way.

#### 5.3.3. Modeling Ladders

The last step entails approximating the ladder by a set of lines, which takes into account the geometrical features of ladder. The structure of the ladder can be defined by the following properties:

- the rungs are parallel;
- the stiles are parallel;
- the rungs and stiles are orthogonal.

**Figure 17:**
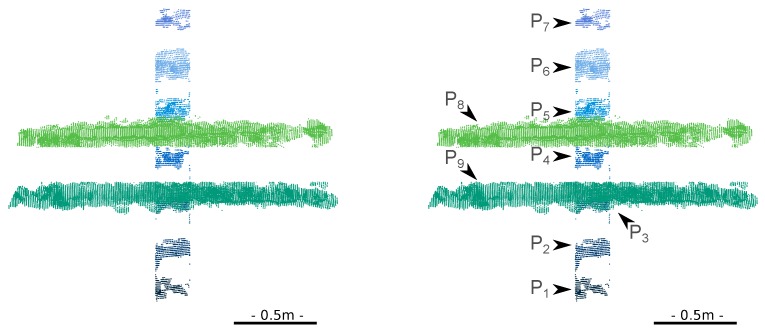
(**Left**) The 2D ladder point cloud is first split into two stile point clouds and seven rung point clouds; (**Right**) The stiles are rotated, and only one direction has to be adjusted on the data.

The orthogonality can be exploited as shown in Figure 17, by applying a 90° rotation to the stile point clouds. Then, only one direction has to be estimated. When the second condition is not satisfied (e.g., for the top part of the flared ladder), the stiles are simply not considered in the estimation.

In [25], we proposed a robust regression technique, that will be recalled below, to model the ladders using the three above assumptions. In the present paper, we propose to use an even more constrained ladder model by introducing *a priori* information about distances, namely:

- the inter-rungs distances, $\delta_{j}$,
- the inter-stile distance, $\delta_{s}$,

which may be estimated from a TLS survey of the ladder; see Figure 14.

**Figure 18:**
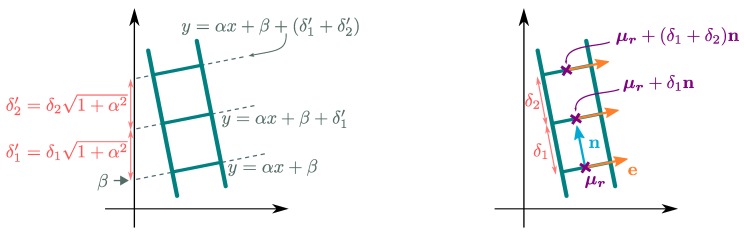
Simultaneous affine (**Left**) and orthogonal (**Right**) regressions.

The linear regression analysis can be performed using either affine or orthogonal regression. Affine regression involves models of the form $y_{i}=\alpha x_{i}+\beta$ and can easily be adapted to the simultaneous robust fitting of multiple lines [26,27]. In our case, the slope *α* is the same for all components thanks to the parallelism constraint. It would be possible to introduce the inter-distance prior by modifying the model as (for the *j*-th rung):

(12) $$y_{i}=\alpha x_{i}+\beta +\sqrt{1+\alpha^{2}}\;\overset{j}{\sum_{k=1}}\delta_{k}$$

(see Figure 18-left). However, this leads to a non-linear relationship with respect to *α*. If the axes provided by the robust estimation of the ladder plane are exactly vertical and orthogonal with respect to the ladder, then α=0, and the problem is reduced to the regression of one intercept, *β*. However, we prefer to avoid such an assumption by considering the orthogonal regression framework.

#### 5.3.4. Orthogonal Simultaneous Fitting of Rungs and Stiles

The model underlying orthogonal linear regression is similar to Equation (5), except that a single vector (the one that supports the straight line) is considered: $x_{i}≃\mu +ea_{i}$; and that $a_{i}$ is a scalar. The robust orthogonal linear regression algorithm is then straightforwardly adapted from the one summarized in Appendix A.

Let us denote by $P_{j}={\left\{x_{\mathrm{ji}}\right\}}_{i=1...N_{j}}$ each of the *R* point clouds corresponding to rungs and the S=2 point clouds corresponding to the side rails, with $\sum_{j=1}^{R+S}N_{j}=N$. Due to the parallelism and orthogonality conditions, the orthogonal simultaneous fitting algorithm is reduced to the estimation of a single direction, e and R+S centroids, $\mu_{j}$. In other words, the underlying model is:

(13) $$x_{\mathrm{ji}}≃\mu_{j}+ea_{ji}$$

The solution of the associated LS problem is given by the sample means, for the centroids:

(14) $$\mu_{j}=\frac{1}{N_{j}}\sum_{i=1}^{N_{j}}x_{\mathrm{ji}}$$

and for the common direction e, by the first eigenvector of the global covariance matrix:

(15) $$C=\frac{1}{N}\sum_{j=1}^{R+S}\sum_{i=1}^{N_{j}}\left(x_{\mathrm{ji}}-\mu_{j}\right){\left(x_{\mathrm{ji}}-\mu_{j}\right)}^{⊤}$$

The robust counterpart of this algorithm is derived by alternating computations of the weights, $b_{ji}$, and weighted LS estimations of $\mu_{j}$ and e; see Appendix B.

In Table 2, we show the distances between rungs that were estimated from TLS and MSS data with the proposed method and a commercial software (3DReshaper${}^{®}$). Note that our method fit the rungs and styles simultaneously, while they must be dealt with separately with the commercial software. Moreover, the proposed algorithm exploits orthogonality and parallelism constraints. The TLS data have been obtained in laboratory conditions and can then be considered as almost noiseless. In such a favorable situation, both methods perform well, and we observe differences of 1 mm maximum. The distances obtained by our method will be considered as a reference in what follows. Unlike TLS data, MSS data are very noisy and contain outliers. As shown in Figure 16d, the performance of the commercial software collapses in that case, and since the rungs are not parallel in the resulting model, inter-rung distances cannot be evaluated. In contrast, our method is robust on this dataset. The fourth column of the table shows that the maximum difference with the reference is 28 mm and that most errors are less than 10 mm (which also corresponds to the mean absolute difference, the median absolute difference being 5 mm). One may note that these results are better than the ones obtained in [25]. This is due to the fact that in [25], the reference measurements were made by hand, and a constant inter-distance (of 280 mm) was considered. Using a TLS survey provides a better reference.

**Table 2:** Comparison of inter-rung distance ($\delta_{i}$) estimations obtained with our orthogonal simultaneous fitting algorithm (without distance prior) and a commercial solution (3DReshaper${}^{®}$) from laser and sonar point clouds of the ladder. Distances are in millimeters.

	laser (Low Noise, No Outliers)		sonar (Strong Noise + Outliers)	
	Proposed Solution (Reference)	Commercial Software	Proposed Solution	Commercial Software
$\delta_{1}$	279	279 (0)	276 (−3)	n/a
$\delta_{2}$	282	281 (−1)	266 (−16)	n/a
$\delta_{3}$	283	284 (1)	287 (3)	n/a
$\delta_{4}$	278	279 (1)	275 (−3)	n/a
$\delta_{5}$	280	279 (−1)	287 (7)	n/a
$\delta_{6}$	262	261 (−1)	234 (−28)	n/a

This algorithm can be complemented by a second stage (which was not present in [25]), in which the distance priors are fully taken into account. Since the inter-rung distances $\delta_{j}$ are known (third column of Table 2), the centroids of the rungs are related, as illustrated in Figure 18 (right), so only two parameters, $\mu_{r}$ and e, must be estimated. In fact, a third parameter, $\mu_{s}$, must be taken into account because the stiles are independent from the rungs in terms of translation, albeit they share the same orientation, up to a 90° rotation. This stage is initialized as follows. A straight line is adjusted on the first *R* centroids, $\mu_{j}$’s, to obtain a first estimate of the axis of symmetry of the ladder. The orthogonal projection of $\mu_{1}$ onto this axis defines $\mu_{r}$. For the stiles, $\mu_{s}$ is defined as the orthogonal projection of $\mu_{R+1}$ on the line of direction e passing through the means of $\mu_{R+1}$ and $\mu_{R+2}$. Then, the algorithm alternately updates e, $\mu_{r}$ and $\mu_{s}$. The complete algorithm is given in Appendix C.

**Figure 19:**
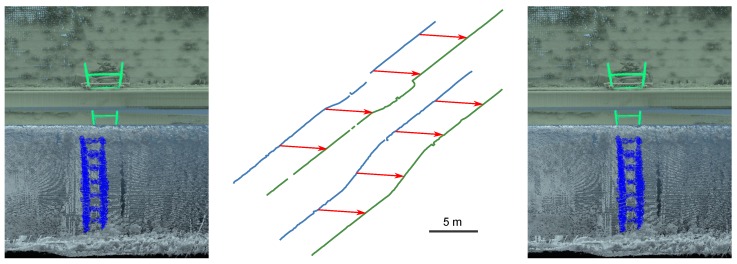
(**Left**) Correction of the vertical translation; (**Center**) Silhouette of the waterline in the TLS and MSS models; (**Right**) Correction of the horizontal translation (final result).

Figure 16c shows an example of robust ladder fitting using the robust orthogonal simultaneous technique with distance priors. One may see that, despite the strong noise level, the ladder is well approximated. It is then possible to correct the vertical gap that was visible in Figure 14 (left). The result is shown in Figure 19 (left).

### 5.4. Horizontal Translation

Once the orientation and vertical translation have been correctly estimated, the last operation consists of estimating the horizontal translation vector.

The 2D silhouette of the waterline along the structure can be extracted on both the TLS and MSS model by intersecting 3D meshes with the plane that corresponds to the water surface (Figure 19 (center)). Finally, we apply a 2D ICP algorithm to estimate the remaining 2D translation vector between both models. After this step, one may see that the continuity of the ladder is restored (Figure 19 (right)).

## 6. Experimental Results

**Figure 20:**
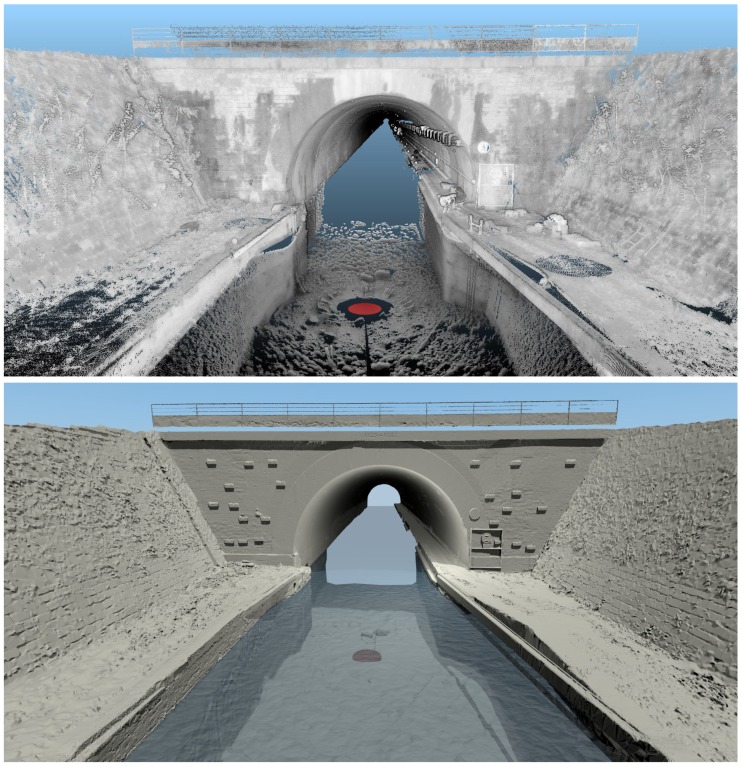
Resulting full 3D geo-referenced model of Niderviller’s canal tunnel entrance. (**Top**) Point cloud visualization; (**Bottom**) Mesh visualization. The red disk indicates the location of the MSS position.

The final 3D, full reference model we obtain with the proposed methodology is shown in Figure 20. We propose two complementary renderings: mesh and point cloud visualization. The latter provides visual information (such as measurement shadows) that may disappear in the mesh visualization, which is smoother. Moreover, some elements (ladders, equipments, packaging) were discarded from the mesh. In the point cloud visualization (Figure 20 (top)), it may be noticed that the above-water and underwater models are visually very satisfying. However, the TLS point cloud seems more homogeneous and denser than the underwater one, which has a lower resolution. Moreover, the MSS model appears more and more grainy as the points are away from the scanning position. For example, many imperfections, both within the MSS model and at the intersection of the models, are visible on canal banks at the bottom of the image. The defects are probably due to the wide footprint of the beam at such a distance from the source at a grazing incidence angle, as already seen in Figure 8. In future experiments, the distances between the MSS positions should be reduced to alleviate these issues.

**Figure 21:**
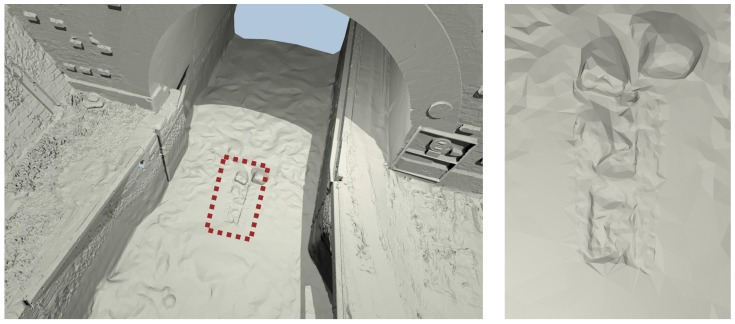
Close-up of the reference model showing two blocks and some structure in the alignment of the cofferdam grooves, which seem similar to the one observed in 2009 at Arzviller (Figure 3).

The construction of the MSS model required many operator interventions for interpreting the elements either as noise or as detail. This task is very difficult without any visual references about the underwater part of the canal. However, just at the entrance of Niderviller’s tunnel, in the alignment of the cofferdam grooves, a detail, which suggests a kind of step, may be seen (Figure 21) and has been conserved as an element of interest. This detail reminds us of the image of Arzviller’s tunnel (see Figure 3) where a step is clearly visible between the cofferdam grooves. Unfortunately, we did not find a similar picture of the entrance of Niderviller’s tunnel, which could confirm the relevance of this detail in the MSS model.

## 7. Conclusions and Future Work

In this paper, we have introduced a robust method to build a full 3D model of a canal tunnel. Data have been collected by TLS and MSS devices. The first issue we identified is due to the differences in spatial resolution and beam width between both devices. MSS data are intrinsically noisy and have a much lower resolution than TLS data. In addition, the angular loss of resolution can be rather strong: the oblong shape of the tunnel induces many grazing incidence angles, and the sonar data are rather coarse at large distances from the scanner device.

The first processing step consists of denoising the MSS data by meshing. More specifically, a coarse-to-fine method, which gradually increases the resolution of the mesh, is applied. Of course, differentiating noise from details is a difficult task in the absence of visual or physical reference, and confusion is unavoidable. The interpretation by an operator is required to determine the appropriate trade-off between noise and details. We believe that acoustic and image processing techniques should be explored to devise more automatic and data-driven denoising methods. In particular, moving least squares, bilateral filtering [28], non-local means filtering [29,30,31] or structure+texture decompositions [32] seem appealing for this task.

A second challenge concerns the co-registration of the point clouds provided by both scanners. The methods for processing TLS data to generate the geo-referenced above-water 3D model are well-known and can be used without any difficulties in our context. On the other hand, handling the MSS point clouds to build the underwater model is much more complicated. In particular, the weak resolution and noisy aspect of MSS data, along with the lack of salient elements along the canal, make classical registration methods, such as ICP, less efficient. To alleviate this difficulty, the experimental setup must be improved by reducing the distance between MSS positions. An interval of about 5 m would be recommended, instead of 10 meters, as in the current experiment. Moreover, additional targets, such as the ladders that are used in our experiment, could be immersed in the canal. They could make the registration easier, by adding references to both point clouds. Our experiments show that the targets must be carefully chosen and placed on-site: for example, the ladders should be wooden and separated from the canal walls; otherwise, their automatic segmentation becomes problematic.

To obtain a full 3D model of the canal tunnel, registering MSS and TSS data is a crucial point. The lack of overlap between point clouds raises difficulties in our application. To solve this challenge, we proposed a three-step procedure in which, first, geometrical entities are exploited to determine the orientation parameters. The second step uses the ladders to estimate the vertical correction: we introduced a robust method based on M-estimation to simultaneously fit lines on the stiles and rungs of the ladder. We assessed the method by comparing the results of the robust fit with real distance measurements. Distance priors are used to increase the precision of the fit thanks to a reference scan of the ladders. The proposed methodology could be adapted to other manufactured targets. Finally, the silhouette of the waterline is extracted in both models, and a 2D ICP algorithm is applied to estimate the remaining horizontal translation vector. Since the TLS point cloud is bound to a geodetic system, the geo-referencing of the model comes as a by-product of our method, without additional complexity.

This experimentation provides an initial overview of underwater acquisition in canal tunnels and yields promising results. Improvements of the model quality may be expected from a better experimental setup (closer scanning positions, more numerous targets). More automatic and data-driven filtering techniques should help with enhancing the data quality and reducing manual interventions. Furthermore, an experiment in a controlled environment, like a dry dock, as proposed in [5], would allow a fine assessment of the model accuracy. One may foresee that the progress of technology will improve the performances of the acquisition devices. Subsequently, we may expect more accurate models using the proposed methodology. The obtained models should be used as a reference for future acquisitions, either with dynamic underwater acquisitions systems (for assessing mobile mapping solutions) or in static ones (to evaluate the tunnel deformation).

## Appendix

### A. Robust Regression of Planes and Lines

1. Initialize $\mu^{0}$ the centroid and $E^{0}$ vectors defining the plane, and set the iteration index to t=1.
  - $\mu^{0}=\frac{1}{N}\sum_{i=1}^{N}x_{i}$
  - $E^{0}$ is the eigenvectors of $C^{0}$ that match their two largest eigenvalues, where:
    with $C^{0}=\frac{1}{N}\sum_{i=1}^{N}\left(x_{i}-\mu^{0}\right){\left(x_{i}-\mu^{0}\right)}^{⊤}$
2. Compute residuals: $r_{i}^{t}=∥\left(\mu^{t-1}+E^{t-1}a_{i}^{t}\right)-x_{i}∥$ with $a_{i}^{t}=E^{{t-1}^{⊤}}\left(x_{i}-\mu^{t-1}\right)$.
3. Compute auxiliary variables $b_{i}^{t}=\frac{\rho^{\prime }\left(r_{i}^{t}\right)}{2r_{i}^{t}}$ and their sum $S^{t}=\sum_{i=1}^{N}b_{i}^{t}$.
4. Perform a weighted LS estimation to get $\mu^{t}$ and $e^{t}$
  - $\mu^{t}=\frac{1}{S^{t}}\sum_{i=1}^{N}b_{i}^{t}x_{i}$
  - $E^{t}$ is the eigenvectors of $C^{t}$ corresponding to its two largest eigenvalues.
    with $C^{t}=\frac{1}{S^{t}}\sum_{i=1}^{N}b_{i}^{t}\left(x_{i}-\mu^{t}\right){\left(x_{i}-\mu^{t}\right)}^{⊤}$
5. If $\frac{∥\mu^{t}-\mu^{t-1}{∥}^{2}}{∥\mu^{t-1}∥}>\epsilon$ increment t and go to 2, else $\mu^{IRLS}=\mu^{t}$ and $e^{\mathrm{IRLS}}=e^{t}$.

This algorithm can be straightforwardly adapted to the case of line estimation. In that case, only one vector (the direction of the line), e, is sought. The expression of the residual becomes $r_{i}^{t}=∥\left(\mu^{t-1}+a_{i}^{t}e^{t-1}\right)-x_{i}∥$ with $a_{i}^{t}=e^{{t-1}^{⊤}}\left(x_{i}-\mu^{t-1}\right)$. The estimation of the means does not change, and e is given by the eigenvector of C that matches its largest eigenvalue.

### B. Simultaneous Robust Fitting of Lines

1. Initialize $\mu_{j}^{0}$ centroids and $e^{0}$ the direction vector, and set the iteration index to t=1.
  - $\mu_{j}^{0}=\frac{1}{N_{j}}\sum_{i=1}^{N_{j}}x_{\mathrm{ji}}$
  - $e^{0}$ is the eigenvector of $C^{0}$ that matches its largest eigenvalue, where:
    $C^{0}=\frac{1}{N}\sum_{j=1}^{R+S}\sum_{i=1}^{N_{j}}\left(x_{\mathrm{ji}}-\mu_{j}\right){\left(x_{\mathrm{ji}}-\mu_{j}\right)}^{⊤}$
2. Compute residuals: $r_{ji}^{t}=∥\left(\mu_{j}^{t-1}+a_{ji}^{t}e^{t-1}\right)-x_{\mathrm{ji}}∥$ with $a_{ji}^{t}=e^{{t-1}^{⊤}}\left(x_{\mathrm{ji}}-\mu_{j}^{t-1}\right)$.
3. Compute auxiliary variables $b_{ji}^{t}=\frac{\rho^{\prime }\left(r_{ji}^{t}\right)}{2r_{ji}^{t}}$ and the sums $S_{j}^{t}=\sum_{i=1}^{N_{j}}b_{ji}^{t}$ and $S^{t}=\sum_{j=1}^{R+S}S_{j}^{t}$.
4. Perform a weighted LS estimation to get $\mu_{j}^{t}$ and $e^{t}$
  - $\mu_{j}^{t}=\frac{1}{S_{j}}\sum_{i=1}^{N_{j}}b_{ji}^{t}x_{\mathrm{ji}}$
  - $e^{t}$ is the eigenvector of $C^{0}$ corresponding to its largest eigenvalue.
    with $C^{t}=\frac{1}{S^{t}}\sum_{j=1}^{R+S}\sum_{i=1}^{N_{j}}b_{ji}^{t}\left(x_{\mathrm{ji}}-\mu_{j}^{t}\right){\left(x_{\mathrm{ji}}-\mu_{j}^{t}\right)}^{⊤}$
5. If $\frac{∥\mu_{j}^{t}-\mu_{j}^{t-1}{∥}^{2}}{∥\mu_{j}^{t-1}∥}>\epsilon$ increment t and go to 2, else $\mu_{j}^{IRLS}=\mu_{j}^{t}$ and $e^{\mathrm{IRLS}}=e^{t}$.

### C. Simultaneous Robust Fitting of Lines with Distance Prior

1. Initialize $\mu_{r}^{0}$ and $\mu_{s}^{0}$ centroids and $e^{0}$ the direction vector, and set the iteration index to t=1.
  (a) Estimate ${\mu_{j}^{0}}^{\prime }$ centroids and $e^{0}$ the direction vector thanks to the simultaneous robust fitting algorithm; see Appendix B.
  (b) Estimate $\mu_{r}^{\prime }$ the centroid of the fitted line of ${\mu_{j}^{0}}^{\prime }$ with j={1,...,R} (see Appendix A).
    Thus, $\mu_{r}^{0}=\mu_{r}^{\prime }+\left({n^{0}}^{⊤}\left({\mu_{1}^{0}}^{\prime }-\mu_{r}^{\prime }\right)\right)n^{0}$ with $n^{0}$ the normal to $e^{0}$
  (c) Compute $\mu_{s}^{\prime }=\frac{{\mu_{R+1}^{0}}^{\prime }+{\mu_{R+2}^{0}}^{\prime }}{2}$.
    Thus, $\mu_{s}^{0}=\mu_{s}^{\prime }+\left({n^{0}}^{⊤}\left({\mu_{1}^{0}}^{\prime }-\mu_{s}^{\prime }\right)\right)n^{0}$
  (d) Compute $\mu_{j}^{0}$ centroids from:
    - $\mu_{j}^{0}=\mu_{s}^{0}+n^{0}\;\sum_{k=1}^{j}\delta_{k}$ for j={1,...,R}
    - $\mu_{R+1}^{0}=\mu_{s}^{0}$ and $\mu_{R+2}^{0}=\mu_{s}^{0}+n^{0}\delta_{s}$
2. Compute residuals: $r_{ji}^{t}=∥\left(\mu_{j}^{t-1}+a_{ji}^{t}e^{t-1}\right)-x_{\mathrm{ji}}∥$ with $a_{ji}^{t}=e^{{t-1}^{⊤}}\left(x_{\mathrm{ji}}-\mu_{j}^{t-1}\right)$.
3. Compute auxiliary variables $b_{ji}^{t}=\frac{\rho^{\prime }\left(r_{ji}^{t}\right)}{2r_{ji}^{t}}$ and the sums $S_{r}^{t}=\sum_{j=1}^{R}\sum_{i=1}^{N_{j}}b_{ji}^{t}$, $S_{s}^{t}=\sum_{j=R+1}^{R+2}\sum_{i=1}^{N_{j}}b_{ji}^{t}$ and $S^{t}=S_{r}^{t}+S_{s}^{t}$.
4. Perform a weighted LS estimation to get $\mu_{r}^{t}$, $\mu_{s}^{t}$ and $e^{t}$:
  - $e^{t}$ is the eigenvector of $C^{0}$ corresponding to its largest eigenvalue, where:
    with $C^{t}=\frac{1}{S^{t}}\sum_{j=1}^{R+S}\sum_{i=1}^{N_{j}}b_{ji}^{t}\left(x_{\mathrm{ji}}-\mu_{j}^{t-1}\right){\left(x_{\mathrm{ji}}-\mu_{j}^{t-1}\right)}^{⊤}$
  - $\mu_{r}^{t}=\frac{1}{S_{r}}\sum_{j=1}^{R}\sum_{i=1}^{N_{j}}b_{ji}^{t}\left(x_{\mathrm{ji}}-n^{t}\sum_{k=1}^{j}\right)\delta_{k}$ for j={1,...,R}
  - $\mu_{s}^{t}=\frac{1}{S_{s}}\left(\left(\sum_{i=1}^{N_{R+1}}b_{(R+1)i}^{t}x_{(R+1)i}\right)+\left(\sum_{i=1}^{N_{R+2}}b_{(R+2)i}^{t}\left(x_{(R+2)i}-n^{t}\delta_{s}\right)\right)\right)$
5. Deduce $\mu_{j}^{t}$ centroids from:
  - $\mu_{j}^{t}=\mu_{s}^{t}+n^{t}\;\sum_{k=1}^{j}\delta_{k}$ for j={1,...,R}
  - $\mu_{R+1}^{t}=\mu_{s}^{t}$ and $\mu_{R+2}^{t}=\mu_{s}^{t}+n^{t}\delta_{s}$
6. If $\frac{∥\mu_{j}^{t}-\mu_{j}^{t-1}{∥}^{2}}{∥\mu_{j}^{t-1}∥}>\epsilon$ increment t and go to 2 else $\mu_{j}^{IRLS}=\mu_{j}^{t}$ and $e^{\mathrm{IRLS}}=e^{t}$.
